# Section on Practice of Medicine, Materia Medica and Physiology

**Published:** 1881-06

**Authors:** 


### PROCEEDINGS OF SECTIONS.

### FIRST DAY.

SECTION ON PRACTICE OF MEDICINE, MATERIA MEDICA AND PHYSIOLOGY.—Dr. Pepper being absent, Dr. J. A. Octerlony, of Louisville, was called to the chair, Dr. T. A. Ashby, of Baltimore, Secretary, being in place.

Dr. W. C. Wile, Sandy Hook, Connecticut, read a paper upon "Blood-letting as a Therapeutic Measure in Pneumonia." He said that it was with great hesitation that he approached such a subject, but, that in the light of his past experience, he felt it to be his duty to do so. He could not, in the limited time at his disposal, say anything in regard to the etiology or diagnosis of pneumonia, but should confine his remarks to the consideration of a therapeutic measure. He then proceeded to say that, while he knew he was treading upon almost forbidden ground, and that he would meet with great opposition from the profession at large, he felt compelled to urge the importance of the *free use of the lancet* in pneumonia. He cited a typical case of what he called "Acute Catarrhal Pneumonia," in which he had recommended the abstraction of blood in the first stage, but in which, owing to the opposition of the family, he had been unable to carry out the practice he recommended until the disease had advanced to the second stage, but even then with the most marked and immediate favorable results. He entered very fully into the details of this case, as it was one of a class which had proved peculiarly fatal in Connecticut, under any treatment he had adopted; and reported a series of twelve other similar consecutive cases, with like favorable results.

Dr. Pepper, having arrived and assumed the chair, at the conclusion of the reading of Dr. Wile's paper, suggested that the discussion be postponed until two other papers upon this subject, which had been promised, should have been read. Dr. Pepper then called upon Dr. Gross to open the discussion.

Dr. Gross said he should prefer to hear from some other gentleman first, and reserved his remarks for the present.

Dr. John J. Lynch, of Baltimore, remarked that he thought, from Dr. Wile's description of his cases, that he had made a mistake in diagnosis, as they were evidently "*croupous*," and not "*catarrhal*" pneumonia. The Doctor was also probably mistaken in attributing the rapid recovery of his cases to the bleeding, because croupous pneumonia most frequently ends in from two to eight days by sudden crisis, and Dr. W.'s cases would probably have recovered just as soon without the bleeding. Dr. L. admitted that for rapidly extending collateral hyperæmia in pneumonia bleeding might be necessary, and was most efficient, but this was from the merely mechanical effect of the remedy, and in no sense curative. The disease would run its course afterwards in spite of the bleeding.

He hoped he would never see the day when the profession returned to the sanguinary method of treating pneumonia, less than half a century ago, when the average mortality was more than twenty-five per cent., while by the modern, purely rational method, it was, in uncomplicated cases, not more than four or five per cent.

Dr. Whittaker, of Cincinnati, said that he thought too earnest protest could not be made against this revival of venesection at this late period in the history of science. Pneumonia is now known to be a general disease with a local expression in the lungs, just as typhoid fever is a general disease with a local expression in the intestines. There could be no justification of venesection in pneumonia, except upon the theory of a local inflammation. That pneumonia is, however, not a simple local inflammation is proven by the fact that it differs in its thermal and special relations from local inflammations, like bronchitis, and that the local and general symptoms do not correspond in severity. The local signs may be slight and the constitutional severe, and *vice versa*, just as in typhoid fever or any of the exanthemata. Pneumonia prevails at all times and places, and occurs at all ages of life like the various infectious diseases, and modern pathology has put pneumonia in the category of the acute infections. It would be impossible therefore to do anything but harm by the letting of blood at any stage of the disease. How could the letting of blood clear up a consolidated lung by pounds of blood coagulated in its interior? The essayist has, evidently, mistaken the crisis for a cure. The crisis in pneumonia may occur at any time between the fifth and eighth days, and often as early as the third, and neither blood-letting nor any other treatment could possibly cut it short. As for *veratrum viride*, that dangerous cardiac sedative, to which the speaker had alluded, it could do nothing but harm. What advantage is there in checking the force and frequency of the heart, when this increase in force and frequency is only compensatory, and is to be favored rather than checked. Pneumonia is due to a poison entering the blood and affecting the whole body, and no amount of blood-letting could let it out, any more than we can drain out the impurities of a stream with a bucket. Our illustrious professor of surgery—may the luster of his name never grow less—may succeed by the force of his genius in restoring venesection, the lost art as he calls it, to a place in the domain of surgery, but in internal medicine it is hopelessly and irretrievably lost.

Dr. N. S. Davis, of Chicago, said that he had practiced medicine for nearly half a century, and that for fifteen years he had practiced venesection after the regular sanguinary method, and that he must say that he had found the best results from blood-letting in pneumonia, when discriminately employed.

Dr. Octerlony, of Louisville, Ky., said that he did not wish to discuss the general subject of pneumonia, but to offer some remarks in analysis and friendly criticism of Dr. Wile's paper. In the first place, he did not deny that cases are occasionally met with in which blood-letting is indicated and will prove beneficial in relieving venous congestion, but he did not think Dr. Wile's cases sufficiently numerous to establish a law, nor were they reported with sufficient care to make them available.

The first case, which the Doctor designated as *catarrhal pneumonia*, was evidently *croupous* pneumonia. Of the other cases he gave no account of the physical signs, and so had left the Society in doubt as to the diagnosis.

The first stage of the disease was the period during which Dr. Wile would have us bleed, because this was the stage of engorgement; but he (Dr. O.) claimed that even at this period exudation had already occurred, as shown by the physical signs, dullness, absence of vesicular murmur, and the presence of fine crepitant *râles*. Bleeding will not return this exudation to the vessels; this can only be accomplished by a vital process. The mortality in Dr. W.'s cases was 1 in 13—far greater than that resulting from the plan of *promoting the natural progress of the disease and supporting the strength of the patient*. The duration of his cases was not less, but greater, than our knowledge of the natural history of the disease shows to be the rule.

In conclusion, he must insist that the writer had not proved his proposition—viz., that pneumonia is a very fatal disease, nor that the mortality or duration of the disease have been diminished by venesection. On the contrary, it seems to be a well established fact that pneumonia is a self-limited disease. The blood-letting plan of treatment used to give a mortality of 1 in 5. The purely expectant plan, 1 in 13. The plans he had here recommended gave, in Bennett's hands, a mortality of 1 in 36.

Dr. J. H. Claiborne, of Virginia, said that he did not think we could fail to go astray if we followed the teaching of the gentlemen who always recommended bleeding, nor could we fail to go astray if we believe those who tell us never to bleed. The middle course was safest, and we should be guided more by experience than by theory. There was no one method of treatment applicable to all varieties of pneumonia.

Dr. Post, of New York, said that in his opinion no dependence could be placed in statistics, as they were drawn from a class of patients found in hospitals, who had been badly clothed and badly housed and fed all their lives, and such statistics were not reliable guides. We must be guided solely by experience. He could not sympathize with the gentleman who expressed the opinion that the lancet could no longer be used in medicine, for he believed that, when properly and discriminately used, recovery was more rapid after bleeding.

Dr. Coleman, of Vermont, said that theoretically he believed in the lancet in a certain class of cases, but that in practice he did not meet with that class of cases where the blood was being forced into the lungs so rapidly as to produce strangulation, as he was not called in time to see them in that condition.

Dr. Quimby, of Jersey City, said that in all acute inflammatory cases where he used stimulants he lost his patients. Under a modified treatment he had lost not a patient. All cases where he had used the lancet he had treated so on first or second day. He thought we did not often see these typical cases in the very first stages. We can't treat patients as if they were machines. He advocated the free use of the lancet, when properly and timely used.

Dr. Whitney, of New York, said that when he first commenced practice he had felt it to be his duty to bleed all cases of pneumonia. Since then he had, to a great extent, abandoned the use of the lancet; but he thought the mortality had never been so great before as since the abandonment of venesection. He thought we did not bleed half enough.

Dr. Ballou, of Rhode Island, had never used the lancet—that he regretted it—but had often failed to bleed, and regretted it very much.

Dr. North, of Iowa, always carried a lancet; he had never lost a case of pneumonia, and had never yet bled one.

Dr. Jackson, of Virginia, thought it objectionable to speak of blood-letting as a treatment of pneumonia—we ought to speak of it as an adjuvant or auxiliary. It was wrong to let it go abroad among the younger men that we had either advised against or for blood-letting.

Dr. S. D. Gross belonged, he said, to the younger members of the profession. He had listened with deep interest to the remarks made here. Pneumonia was an inflammatory affection, and the lancet, in such affections, was applicable in young, robust, and healthy subjects. Bleeding was not applicable in *all* stages of pneumonia, but only in the commencement, when the disease was in its infancy—not later. We employed bleeding simply as an adjuvant, and under such circumstances we employ it wisely. If, however, we wait until consolidation has taken place, bleeding does harm, no matter what be the form of pneumonia, whether croupous or catarrhal. If bleeding was performed in time, and on proper subjects, it was the great remedy in this disease.

Dr. Lyster, of Michigan, did not think the practice of venesection so good as the theory. We did not bleed in cerebro-spinal meningitis, because we knew we should lose our patients; but we used the supporting treatment. He asked what was the difference between the two inflammatory conditions? He was surprised at so much advocacy of the lancet in pneumonia, and would like to see it dispensed with.

Dr. Martin, of Massachusetts, trusted that it might go out to the world that the American Medical Association sanctions the *occasional* use of the lancet, at least.

Dr. Wile, of Connecticut, in closing the discussion, expressed his gratitude for the courteous manner in which his paper had been discussed, and he accepted from the gentlemen their diagnosis of his cases. He felt delighted to learn that pneumonia was such a pleasant disease to treat as those gentlemen who proscribed the lancet seemed to think it. The cases he had reported were of the class, and the only class, he had met with in Connecticut. He did not wish to be understood as saying that the lancet was the only mode of treating pneumonia, but that it was the only way of treating those cases of pneumonia with which he had come into contact in Connecticut.

On motion of Dr. Gross, adjourned.

SECOND DAY.

SECTION ON PRACTICE OF MEDICINE, MATERIA MEDICA AND PHYSIOLOGY.—Met at Mozart Hall at 3 P. M. Dr. Pepper being absent, Dr. J. A. Octerlony, of Louisville, was called to the chair; Dr. T. A. Ashby, of Baltimore, Secretary, being in place.

Dr. Prentiss, of New York, being absent, at the request of the chairman, Dr. King, of Washington, read Dr. Prentiss' paper, entitled "Is Croupous Pneumonia a Zymotic Disease?" The paper was one of some length, and maintained that croupous pneumonia was a constitutional disease; that the lung affection was purely a local manifestation, and that, therefore, pneumonia must be classed among the zymotic and preventable diseases. The differential diagnosis between croupous and catarrhal pneumonia was dwelt upon, the writer claiming that the two diseases had only one point in common, viz.: That in both there was an inflammation of the lung, and, that, in fact, catarrhal pneumonia was merely a local disease, while croupous pneumonia was a constitutional disease with a local (lung) manifestation. The specific cause of croupous pneumonia yet remained undiscovered; the disease must run its course, and that treatment must therefore be symptomatic. He also urged the importance of giving croupous pneumonia its proper classification in mortuary statistics.

Dr. W. C. Dabney read a paper on "Nature and Treatment of Pneumonia," with conclusions as follows:

Two views are held as to nature of pneumonia—one that it is a specific fever; the other, that it is a local inflammation of which the fever is symptomatic.

Arguments in favor of the first view:

1. The disease ordinarily commences with a chill.

2. The constitutional disturbance is often out of proportion to the local disease.

3. The disease usually runs a definite course, and is self-limited.

4. The disease occasionally occurs as an epidemic.

Arguments in favor of second view:

Traumatic pneumonia is precisely similar to the idiopathic form.

The indications of treatment in the first stage are: (1) To lessen the amount of blood in the lungs, and to check, as far as possible, the extension of the inflammation. (2) To reduce the temperature; and (3) to relieve pain.

To fulfill the first indication, blood-letting, diaphoretics, saline purgatives, and cardiac sedatives are to be employed.

To fulfill the second indication, quinine is the most important agent.

To relieve pain, sinapisms, wet and dry cups, and opiates may be resorted to.

In the second stage, the indications are: (1) To lessen the consistency of the fibrinous exudation; and (2) to prevent over-distension of the heart.

To fulfill the first indication, alkalies, especially carbonate of ammonia, are to be employed.

To fulfill the second, alcoholic stimulants are especially useful. Digitalis may also be used with advantage. If so much respiratory surface is involved as to interfere seriously with respiration, oxygen gas should be used.

In the third stage, tonics, a nutritious diet, etc., are advisable.

The Chairman said that the reading of these two valuable papers having been finished, he would call for the discussion on this, one of the most important subjects now before the medical profession.

Dr. Lynch, of Baltimore, opened the discussion, and said that the various arguments which go to prove that croupous pneumonia was a zymotic disease had failed to convince him that such was the fact. If it was a zymotic disease, then the treatment was a matter of little or no importance, and should be merely symptomatic, while if it was a local disease, the treatment was of great importance. He believed that it was often aborted by the use of the proper remedies, and had often seen the so-called defervescence produced in a few hours by the use of veratrum viride.

Dr. Whittaker classed himself with those who believed croupous pneumonia to be an acute infectious disease (not, of course, contagious). One point in the differentiation of this disease had not been brought out, viz.: Catarrhal pneumonia attacked old people principally, while croupous pneumonia was confined to young ones. This, he thought, was a point of importance in diagnosis of cases.

Dr. Burlington, Vermont, did not think we could begin to treat the disease intelligently until we had determined its cause.

Dr. Lester, Missouri, had failed yet to hear the argument which convinced his mind that pneumonia was a zymotic disease. He thought, also, that the discussion yesterday proved conclusively that blood-letting was applicable to a very limited number of cases.

Dr. N. S. Davis, Chicago, had never been able to decide that pneumonia was zymotic, but believed it to be local. He rose to protest against the course of reasoning pursued by some members. Even assuming that pneumonia was a zymotic disease and ran a definite course, this was no reason why it might not be cut short, and the physician had no right to fold his hands and do nothing because of such reasoning.

Dr. Lynch, of Baltimore, approved of Dr. Davis' remarks, but thought that, in the present state of our knowledge, we were not justified in using any remedy about which we were uncertain in an attempt to cut it short. In reference to the use of the alkalies in pneumonia, he believed them to be valuable, particularly carbonate of ammonia, in certain stages of the disease, but not in the initial or even second stage.

Dr. Octerlony said that it seemed to be settled by discussion that if pneumonia was a zymotic disease it could not be cut short, but if local, it could. Pneumonia, he thought, was a self-limited disease. In regard to the zymotic character of the disease, he must confess, his mind had not been convinced; thought it local; wished to call attention to the danger of death from heart-clot.

He thought carbonate of ammonia useful in other stages beside the third.

Dr. Ball, of Ohio, thought the idea of pneumonia being a self-limiting disease, and founding treatment upon that idea, to be exceedingly erroneous, and calculated to do great harm. Thought the disease could be cut short, and called attention to the method of treatment by repeated emetics early in the disease, and claimed that it was valuable in aborting the disease.

Dr. McCaul, of Michigan, thought that in his section, venesection was not admissible, and that cardiac sedatives must be used with extreme caution. Did not think the disease was purely zymotic, but thought there was a zymotic condition of blood which tended to produce it.

Dr. Whittaker, of Cincinnati, asked if pneumonia were a local disease, what caused it? certainly exposure did not. We had, he thought, no right to call it a local disease until we knew its etiology.

On motion of Dr. Davis, of Chicago, the discussion upon this subject was closed, and the papers referred to the appropriate committee.

The Secretary read by title a paper by Dr. Robertson, of New York, who was absent, entitled, "Nature and Treatment of Pulmonary Phthisis." Referred to committee.

The time until adjournment was then occupied in the reading, by Dr. Bulkley, of New York, of a very valuable and interesting paper on the "Diet and Hygiene of Eczema."

THIRD DAY.

SECTION OF PRACTICE OF MEDICINE.—Dr. L. Duncan Bulkley, of New York, read a paper upon "The Diet and Hygiene of Eczema." The paper was based almost wholly upon the experience of the writer, inasmuch as very little thought had been bestowed on the subject by others, and no explicit instructions were to be found in relation to the matter in the text-books or writings on the subject.

After calling attention to the effect which certain articles, as shell-fish, strawberries, bananas, etc., produced upon the skin of hose who were peculiarly disposed thereto, in causing erythema and urticaria, and also the well-recognized eruptions from iodide and bromide of potassium, quinine, etc., he stated that in like manner articles of food and modes of life surely did produce cutaneous changes, and that the genesis and obstinacy of many cases of eczema, rested upon errors which existed in nutrition from faulty diet and hygiene. Give perfect nutrition, and eczema disappears. The errors were not always apparent, and often required to be searched for.

In regard to the true meaning of dieting in this connection, he did not intend thereby a starvation process, as too often supposed, with an idea of starving out the disease, but his definition of diet was as follows: "*Such a regulation of the quantity and quality of the food and drink taken, its mode of preparation and time and method of consumption, as shall conduce to the restoration and maintenance of health.*"

In the further development of the subject, he considered separately each element of this definition, calling attention to the errors constantly found in patients suffering from eczema. During the nursing period, too frequent feeding was a source of trouble, the breast should not be given to the child each time it cried from the irritation of the disease. He also called attention to the very great importance of securing healthy breast-milk, and stated that attention should be paid even more to the mother than to the child; if she has constipation and dyspepsia, if her secretions from the bowels, kidneys, etc., are not normal, that from the breast was not normal, and could provoke disease in the child. Ale, beer, excessive use of tea in the mother, and other troubles, as even debility, could excite and continue the disease in the child. He also called attention to the error of feeding children at the breast, or those of older years, with the food of adults, and stated that he daily found the grossest errors of diet in eczema patients. The nurses, to whom so much is often committed, are from the lower walks of life, and know nothing of the laws of health. All these points must receive careful attention from the physician in order to obtain success in obstinate cases.

In the eczema of older life, he called attention to the general disuse of fat found in these subjects, and believed that this was a very important point in the management of these cases; but the fat taken should be in healthy form, and its digestion secured; pasty articles in which fat was found were not desirable, and should be avoided in eczema. Ale, beer and wine were also interdicted; tea and coffee in moderation were not injurious, but tea in excess he had frequently recognized as an element of injury in eczema patients.

Many other points were stated in regard to special articles of food, and also in reference to " its mode of preparation and time and method of consumption," and stress was laid upon the necessity of all these homely subjects receiving the careful attention of the physician, inasmuch as the laity were grossly ignorant in the matter.

In regard to hygiene, attention was called to proper and sufficient exercise, bathing, etc., indeed to every item which could in any way conduce to the health of the patient.

Dr. Whittaker, of Cincinnati, read a paper on the Treatment of Diphtheria, taking the ground that diphtheria was first a local and afterwards a general disease, that it is only when the epithelial barrier is broken down that the blood and body become infested. The essayist maintained that the poison passes over into the blood little by little, new quantities reinforcing the first installments until the blood is super-saturated with the disease. The treatment therefore resolved itself into treatment of the poison at the local depot and relative neutralization of virus in the blood. The efficacy of the antiseptics quinia, salicylic acid, and the benzoates was next discussed, and the experiments with the latter of Buckholtz and Graham Brown, whereby it was shown that saturation of the blood with the benzoates renders inoculation impossible. The essayist next maintained that although we could not kill the germs of the disease in the throat, we could so condense its mucous membrane as to make it a dam to the influx of the disease. To effect this purpose the essayist recommended the per-sulphate of iron in full strength, applied well up behind the velum palati. Though the per-sulphate of iron was one of the oldest recommendations of practical medicine in the treatment, the author believed its occasional inefficacy and present comparative disuse to be due to the dilution of the solution. The author knew very well the fallacy of basing conclusions upon experience, and mentioned the fact that he had never had a fatal case since the use of this treatment, and to state that he had never seen any accidents incidental to the treatment.

The writer did not believe in the contagiousness of dysentery, nor did he think that bacteria were always present in the evacuations. Neither did he think that it was due to malaria or scorbutus, though either one might complicate it. In spite of Dr. Austin Flint's forty-four cases (and the writer deemed this number too small for a basis of belief), he thought dysentery might occur at any season. He does not think that there is any clinical difference between catarrhal and epidemic dysentery. The tendency of the disease, when influenced by treatment, was towards recovery.

The first indication of treatment was to get the bowel into a proper condition to receive medication. 2. Diet; 3. Rest; 4. Demulcents; 5. Control of morbid processes.

Ipecac was, he thought, unnecessary in the majority of cases met with in common practice. Ergot, coto bark, injections, suppositories, etc., all received due mention.

The paper was referred, without discussion, to Committee on Examination.

A paper by Dr. I. E. Atkinson, on the Production of Albuminuria by Use of Iodide of Potassium in Syphilitic Disease, was an exceedingly valuable contribution, showing great research, and provoked a very interesting discussion in which many members took part.

Dr. Whittaker, of Cincinnati, did not think anything more than a transient albuminuria would be produced by the use of the potassium, but thought it was, more likely, caused by renal cirrhosis. It would certainly be more in accordance with our present knowledge of pathological anatomy to attribute these cases to the disease and not the remedy.

Dr. Octerlony wished to emphasize the fact that albuminuria was not a disease, but merely a symptom—one that might occur in perfectly healthy persons.

Dr. Lynch, Baltimore, was surprised to learn that iodide of potassium was accused of producing albuminuria; always used iodide of potassium in half drachm doses in Bright's disease, and always with happy effect.

Dr. Dillard, of Lynchburg, corroborated the statement of Dr. Lynch.

Dr. Atkinson said that he did not bring any charges against the use of iodide of potassium in general, but merely made an inquiry into one special use of it, with the view of showing what he thought was one of its disadvantages. He regarded iodide of potassium as one of our most valuable remedies, and when it produced changes in the mucous membrane of the kidneys, he attributed it to an idiosyncrasy on the part of the patient.

On motion, the paper was referred to the Committee on Examination.

Dr. Henry A. Martin, of Boston, read a paper upon the Variola Vaccinię and Variola Equinię in Massachusetts.

In this paper the Doctor reported a series of cases of cow-pox (which were, so far as he knew, the first authentic cases reported in this country), which were of great interest. He also reported a case of variola equinæ, the seventh authentic case since the days of Jenner. In conclusion, the Doctor moved that this Section recommend to the Association that a committee be appointed to visit these various farms and investigate the whole subject of bovine virus, as it was one subject to many and great abuses. Carried.

*The Materia Medica of the Future* was the title of a paper by Dr. F. E. Stewart, of New York. He maintained that physicians should know the methods of manufacture of drugs. The profession is under many obligations to pharmacy for the elegant preparations of drugs. It is the physician's prerogative to treat the sick, and to dictate how his medicine should be prepared. The code of ethics of the profession prohibits the prescription of secret formulæ, or the holding of a patent on an instrument. But trade has so affected pharmacy by patents, copy-rights, trade-marks, secret formulæ, etc., that a physician is obliged to stultify himself to gain the advantages of many of the improved forms of medicine. Either the code should be altered, or the methods of trade should be changed to meet the requirements of the code. If the patent system be admitted by the profession, the protection by law should be limited, and under the same restriction as applies to other trades, and the exact working formulæ made known; or else no druggist should be allowed to prescribe for the sick, and that no physician should prescribe patent medicine whose nature was unknown.

The paper was referred to the Publishing Committee.

The following resolution, offered by Dr. Dunster, was adopted:

"*Resolved*, That the spirit of the code of ethics forbids a physician from prescribing a remedy controlled by a patent, copyright, or trade-mark. This however shall except a patent upon a process of manufacture or machinery, provided the patent be not used to prevent legitimate competition; and shall also except use of a trade-mark used to designate a brand of manufacture: provided that the article so marked be accompanied by working formulæ, duly sworn to, and also by a technical, scientific name under which any one can compete in manufacture of same."

Dr. Upshur, of Richmond, Virginia, read the report of a case of paralysis of motion of both upper extremities in which the prominent symptoms were complete paralysis of motion of both upper extremities *except in the hands*, intense pain in the shoulders on any attempt to lie down, and inability to sleep. The attack was ushered in by convulsions epileptic in character.

Referred to Committee.

